# The Primary Cilium on Cells of Developing Skeletal Rudiments; Distribution, Characteristics and Response to Mechanical Stimulation

**DOI:** 10.3389/fcell.2021.725018

**Published:** 2021-08-20

**Authors:** Claire A. Shea, Paula Murphy

**Affiliations:** Trinity College Dublin, The University of Dublin, Dublin, Ireland

**Keywords:** skeletogenesis, skeletal development, primary cilia, chondrogenesis, morphogenesis, mechanical regulation, mechanosensory

## Abstract

Embryo movement is important for tissue differentiation and the formation of functional skeletal elements during embryonic development: reduced mechanical stimulation results in fused joints and misshapen skeletal rudiments with concomitant changes in the signaling environment and gene expression profiles in both mouse and chick immobile embryos. Despite the clear relationship between movement and skeletogenesis, the precise mechanisms by which mechanical stimuli influence gene regulatory processes are not clear. The primary cilium enables cells to sense mechanical stimuli in the cellular environment, playing a crucial mechanosensory role during kidney development and in articular cartilage and bone but little is known about cilia on developing skeletal tissues. Here, we examine the occurrence, length, position, and orientation of primary cilia across developing skeletal rudiments in mouse embryos during a period of pronounced mechanosensitivity and we report differences and similarities between wildtype and muscle-less mutant (*Pax3*^*Spd/Spd*^) rudiments. Strikingly, joint regions tend to have cilia positioned and oriented away from the joint, while there was a less obvious, but still significant, preferred position on the posterior aspect of cells within the proliferative and hypertrophic zones. Regions of the developing rudiments have characteristic proportions of ciliated cells, with more cilia in the resting and joint zones. Comparing wildtype to muscle-less mutant embryos, cilia are shorter in the mutant with no significant difference in the proportion of ciliated cells. Cilia at the mutant joint were also oriented away from the joint line.

## Introduction

Embryonic movement is important for correct skeletal tissue differentiation where immobilization leads to characteristic abnormalities of rudiment shape, joint formation and ossification in mouse and chick embryos, as well as corresponding changes in the expression of regulatory genes and signaling pathway activity (Nowlan et al., 2008, 2012; Kahn et al., 2009; Roddy et al., 2011; Rolfe et al., 2014, 2018; Singh et al., 2018). There is clear evidence that the physical extra-cellular environment is capable of influencing cell behavior, including gene expression (Wang et al., 2009; Mammoto and Ingber, 2010; Aragona et al., 2013), however, no specific mechanism has been shown to conclusively transduce the mechanical stimuli generated by embryo movement. Identification of the mechanoregulatory mechanisms at play is essential for understanding how mechanical stimulation plays a role in the patterning of skeletal tissues.

The primary cilium is a non-motile, microtubule-supported organelle that often projects outward from the cell surface, allowing sensation of the mechanical environment (Singla and Reiter, 2006; Malone et al., 2007; Deren et al., 2016; Spasic and Jacobs, 2017b). It is present on diverse cell types in developing and mature tissues and has been studied during organogenesis of the kidneys, nervous system, heart, and skeleton (reviewed in Gerdes et al., 2009; Fry et al., 2014; Tao et al., 2020). The membrane encapsulating the cilium axoneme is distinct from the adjacent cellular membrane, with localized membrane-bound receptors and other cilium-specific proteins (reviewed in Jensen and Leroux, 2017). In multiple contexts, primary cilia act as signaling centers for the Hedgehog (Hh), Wnt, and calcium signaling pathways, with pathway components localized to the cilium (Goggolidou, 2014; Delling et al., 2016; Bangs and Anderson, 2017; Elliott and Brugmann, 2019). The location and structure of the cilium are ideal for responding to extra-cellular stimuli and mechanical forces. The axoneme projects into the extra-cellular space, potentially interacting with components of the extra cellular matrix as well as ligands or other diffusible molecules. It can also respond to mechanical forces such as fluid flow, tension, and compression, as shown in mesenchymal stem cells (MSCs), and cells of the mature endothelia, kidney, liver, and bone (reviewed in Hoey et al. (2012a); Spasic and Jacobs, 2017b). During development, the mechanosensitivity of primary cilia to fluid flow is crucial in establishing asymmetry at the embryonic node, as well as calcium signaling during kidney development; inhibition of ciliary function results in patterning defects and polycystic kidney disease (reviewed in Nonaka et al., 2002; Bisgrove and Yost, 2006).

Several studies indicate a role for primary cilia as mechanosensors during skeletal development. Primary cilia on MSCs were shown to promote osteoblastic differentiation and bone formation by sensing fluid flow (Hoey et al., 2012b), while reduction in cilia length or number may be necessary during chondrogenesis and proliferation (Tummala et al., 2010; reviewed in Yuan et al., 2015; Thompson et al., 2017). In established skeletal tissues, cilia act as mechanosensors and are crucial for the maintenance of healthy articular cartilage and bone (Nguyen and Jacobs, 2013; Ruhlen and Marberry, 2014; Kitami et al., 2019). Cilia are vital to correct Hh signaling in the limb buds of mouse and chick embryos, leading to misspecification of digits and polydactyly when cilia are absent or non-functional (Bangs et al., 2011). The absence of cilia from mesenchymal condensations (under Dermo1-Cre) or cartilage (Col2aI-Cre), or global mutations of ciliary genes, result in limb shortening, disorganized chondrocytes, craniofacial abnormalities, hypomineralization, and dwarfism, suggesting altered cell signaling that impacts skeletal differentiation (Koyama et al., 2007; Song et al., 2007; Tao et al., 2020). Yuan and Yang (2015) showed that primary chondrocytes derived from postnatal cilia-deficient mice had deficient Hh signaling and enhanced responsiveness to canonical Wnt signals. Components of both the Hh and Wnt pathways are altered under reduced mechanical stimulation (Rolfe et al., 2014), and co-ordination of these signaling pathways by cilia in skeletal tissues raises the possibility that cilia are involvement in transducing mechanical signals in this context.

Cilia orientation and length have been examined in post-natal growth plate cartilage and adult articular cartilage (McGlashan et al., 2007; Ascenzi et al., 2011). Cilium orientation is altered in the disorganized growth plates of conditional Smad1/5 knockouts and ciliary knockouts (Ascenzi et al., 2011). The length of the cilium has implications for mechanosensitivity, cell signaling and the capacity of the cilium to deflect (Spasic and Jacobs, 2017a; Ehnert et al., 2017). Despite their proven necessity for normal skeletal development, characterization of cilia in the developing rudiments and joints is lacking in the literature and is an important step in assessing the possible contribution of cilia to the mechanoregulation of skeletal development.

In this work, we characterize primary cilia in the developing skeleton at a key developmental stage for mechanosensitivity, examining wildtype and muscle-less (immobile) mouse embryo skeletal rudiments (*Pax3*^*Spd/Spd*^) (Kahn et al., 2009; Rolfe et al., 2014). An important prerequisite for investigating a role for cilia in specific cell types or regions of the developing limb skeleton is the detection of cilia on those cells. Therefore, a description of cilium occurrence, length and orientation across regions of the developing skeletal rudiments and a comparison between wildtype and immobile embryos during the period of heightened sensitivity is presented.

## Materials and Methods

### Animals and Tissue Preparation

Heterozygous *Splotch delayed* (*Pax3*^*Spd*/+^) (Vogan et al., 1993) (Jackson Laboratories) mice were bred and euthanized under license from the Irish Medicines Board/Health Products Regulatory Authority. Embryos were harvested at embryonic day (E) 14.5, staged (Theiler, 1989), fixed in 4% PFA, dehydrated to methanol and stored at −20°C. Genotype was confirmed by PCR (Keller-Peck and Mullen, 1997).

Dissected wildtype and *Pax3*^*Spd*/Spd^ forelimbs at Theiler Stage (TS)23 (wildtype *n* = 4; mutant *n* = 3) were rehydrated to PBS, equilibrated to 30% sucrose, rapidly frozen, and stored at −80°C. Longitudinal cryosections (5–10 μm) were prepared, collected on SuperFrost Plus slides (VWR, Radnor, PA, United States) and stored at −20°C.

### Immunolocalization of Cilia

Enzyme-mediated antigen retrieval was performed in 20 μg/mL Proteinase K in 0.1M Tris–HCl for 10 min at 37°C. Samples were blocked in 5% goat serum in TBST. Primary antibodies for two types of tubulin localized to the primary cilium (acetylated α-tubulin (Sigma T7451; 1:200) at the axoneme and γ-tubulin (Sigma T5192; 1:200) at the cilium base) were applied in blocking solution, and incubated overnight at 4°C. Slides were incubated in secondary antibody [Invitrogen A11031 goat anti-mouse Alexa Fluor 568 (1:200), and Invitrogen A21206 donkey anti-rabbit Alexa Fluor 488 (1:250)] in blocking solution and mounted in ProLong Gold Antifade Reagent with DAPI (Life Technologies).

**FIGURE 1:**
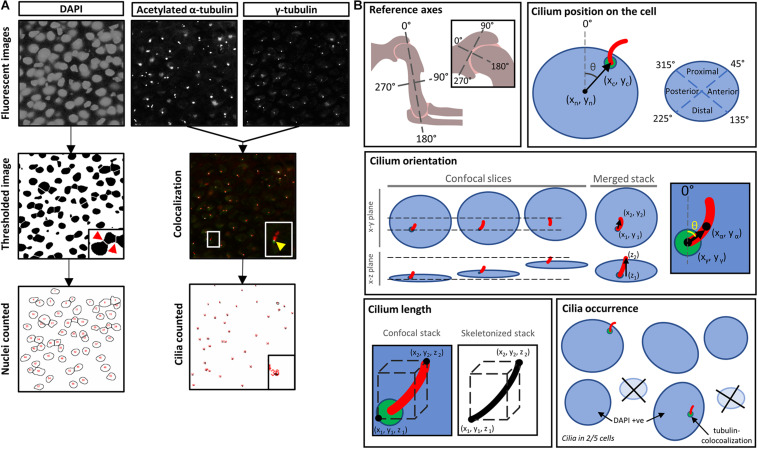
Analysis of cilium characteristics. **(A)** Images of immunostained nuclei (DAPI) and cilia (acetylated α-tubulin and γ-tubulin) captured, processed separately, and recombined. To count nuclei, images were processed as follows (with corresponding ImageJ functions indicated): the image was smoothed (Gaussian Blur; sigma = 3), converted to binary (Threshold), cleared of noise (Despeckle); nuclei were then separated (Watershed) and counted (Analyze Particles; minimum size = 20 μm^2^). In parallel, α- and γ-tubulin images were merged and converted to a binary image of overlapping staining (Colocalization); regions of overlap (indicating cilia) were counted (Analyze Particles). **(B)** Illustrative overview of parameters measured, as detailed in the text. For *cilium position on the cell* and *cilium orientation* analyses, images were aligned to the longitudinal axis of the humerus (*reference axes*).

Images were collected using a fluorescent microscope [Olympus DP72 camera and CellSens software (v1.6)] with 40× objective, or a confocal microscope (Leica TCS SP8, Leica-Microsystems, Germany) with Leica Application Suite software (LAS v5.1); 40× or 63× objective), across nine regions of the developing rudiments and associated joints. Images were imported into ImageJ (version 1.8.0, National Institutes of Health, Bethesda, MD, United States), and cropped to regions of interest (ROIs) (100 μm by 100 μm). Grayscale images (Split Channels function, ImageJ) were used for analysis (Figure 1A).

### Quantification of Cilia Properties

To determine the proportion of ciliated cells within an ROI, cells and cilia were counted in ImageJ (Analyze Particles); DAPI + ve = no. of cells (minimum size, 20 μm^2^); colocalized tubulin staining = no. of cilia (Figure 1A). The co-localization of multiple antibodies to detect cilia reduced the likelihood of false positives from α-tubulin staining of mitotic spindles, or γ-tubulin basal body staining during cell division (Bangs et al., 2011).

A set of reference axes were fitted to the processed images, where 0°–180° is the proximal-distal long axis of the humerus (Figure 1B). The reference axes for the shoulder joint were adjusted so that 0°–180° was perpendicular to the joint line. The coordinates of each detected cilium and nucleus center were calculated in ImageJ, and used to calculate position of emergence on the cell, which was categorized to one of four quadrants (Figure 1B).

Confocal z-stacks (stack size 4–5 μm; z-step 200 nm; xy resolution 120 or 240 nm) were merged in ImageJ (Z-projection), with maximum pixel intensity. To measure cilium orientation, the x- and y-coordinates at the base of the cilium [γ-tubulin (x_γ_, y_γ_)], and at the center of the axoneme [α-tubulin (x_α_, y_α_)] were used to calculate the angle θ (Figure 1B).

Cilium length was calculated using confocal z-stacks of images and applying the Skeletonize3D plugin in ImageJ, which removes pixels from the edges of detected objects, yielding a single-pixel skeleton (Lee et al., 1994), which was then measured (Analyze Skeleton, ImageJ) (Figure 1B). To verify accuracy, this method was compared to manual cilia length measurement using the ImageJ line tool on maximum-intensity Z-projected images, calculating the Euclidean distance between the first and last z-slices in which the cilium was visible (Figure 1B). Cilia extending outside the z-stack were eliminated from analysis. Measurements were comparable across these methods although the automated method is viewed as more accurate as it accounts for cilium shaft curvature.

### Data Plotting and Statistical Analysis

The R package CircStats (Agostinelli and Lund, 2011) was used to plot rose histograms and to calculate circular statistical analyses (circular mean, variance and concentration; Kuiper’s test of uniformity; and Mardia-Watson-Wheeler uniform scores test). One-way ANOVA and chi-squared tests were calculated in base R.

## Results

### Primary Cilia Have Characteristic Properties in Different Regions of Developing Skeletal Rudiments

**FIGURE 2:**
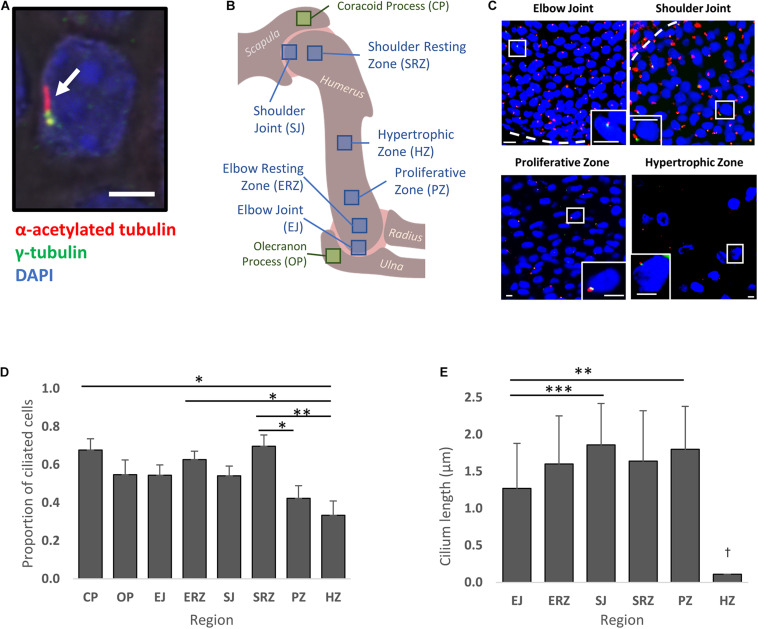
Cilia are present on chondrocytes throughout the normal developing skeletal rudiment at TS23, with some variation in the proportion of ciliated cells and length of cilia detected by region. **(A)** Representative image (confocal) of a primary cilium (white arrow) on a chondrocyte within the developing humerus at TS23; scale bar is 2 μm. **(B)** Schematic of the developing humerus and associated joints, with sampled territories indicated by blue (within the humerus) and green boxes. **(C)** Representative fluorescent images of cilia in selected regions of the developing humerus; scale bar is 10 μm. Inset boxes show zoomed images of representative ciliated cells. Dashed lines indicate the joint line. **(D)** Proportion of total cells (DAPI + ve) with primary cilia detected (α and γ tubulin + ve) by region (abbreviations as indicated in **B**). **(E)** Cilium length by region. Cilia in the hypertrophic zone (HZ) were much shorter; precise measurement was not possible by the approach used but was estimated to be approx. 0.11 μm (†) (not analyzed statistically). Significance in **(D,E)** calculated using one-way ANOVA with *post hoc* Tukey’s tests; ^∗^*p* < 0.05, ^∗∗^*p* < 0.01, ^∗∗∗^*p* < 0.005. *N* = 4 independent specimens for occurrence measurements; *n* = 3 for length measurements.

Primary cilia were unambiguously identified through co-localization of acetylated α-tubulin (axoneme) and γ-tubulin (cilium base) (Figure 2A). Cilia were detected in the resting, proliferative and hypertrophic zones of the developing humerus, at shoulder and elbow joints, and in morphological features of adjacent skeletal rudiments (olecranon and coracoid processes) of wildtype embryos at E14.5 [Theiler Stage (TS)23] (Figures 2B,C). The observed mean proportion of ciliated cells for each zone ranged from 0.33 (hypertrophic zone) to 0.70 (shoulder resting zone) (Figure 2D). The hypertrophic zone had a significantly lower proportion of ciliated cells than the resting zones and coracoid process, where cilia occurrence was highest. The second lowest average proportion of ciliated cells was in the proliferative zone (mean 0.42 ± SE 0.07).

Cilium length was assessed by capturing the full curved length through confocal z-stacks (Figure 2E). Across regions of the developing humerus, cilia lengths varied between 0.1 μm and 4.9 μm. In the hypertrophic zone, cilia were exceptionally short, estimated to be 0.08–0.14 μm, but this was difficult to measure precisely using the skeletonization method; the estimated length was thus derived manually (Euclidean distance through confocal z-stacks) and could not be compared statistically. Cilium length was found to be more consistent across other zones, with the lowest mean observed in the elbow joint (1.27 μm ± SE 0.61); this was significantly lower than both the proliferative zone and the shoulder joint, where cilia were the longest (1.86 μm ± SE 0.56).

**FIGURE 3:**
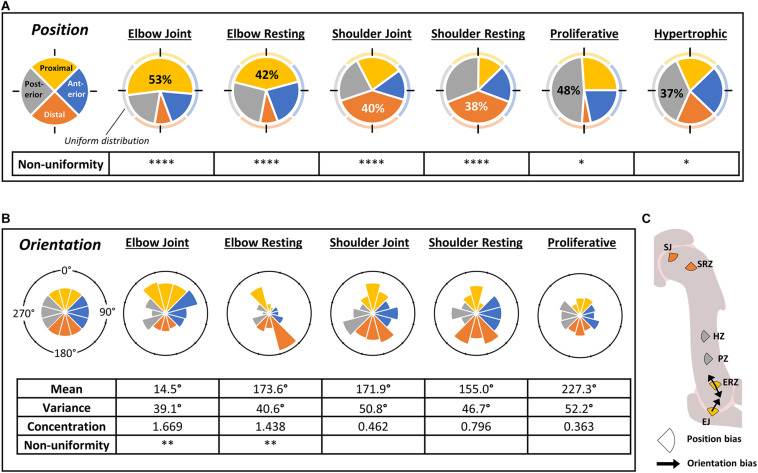
The position of emergence and orientation of cilia on individual cells differs between regions of the normal developing humerus at TS23. **(A)** Pie charts of cilia emergence position on chondrocytes in different regions of the humerus, as indicated; each slice represents the proportion of cilia where the cilium base was located in the given quadrant (as illustrated in Figure 1B); quadrants are color-coded as shown on extreme left where a uniform distribution of 25% in each quadrant is illustrated for comparison. All regions showed significant divergence from uniform distribution (chi-squared test) as indicated. Percentages are noted for the most enriched quadrant. **(B)** Rose histograms of cilium axoneme orientation on chondrocytes in different regions of the humerus, measured from the base to the midpoint of each cilium (as illustrated in Figure 1B (angle θ); bin width = 30°). Angular mean and variance, concentration, and non-uniformity were calculated using the CircStats package in R. High concentration values indicate concentration around certain orientations; low concentration values indicate uniformity. Non-uniformity significance refers to the difference of the observed data from a uniform distribution (Kuiper’s test of uniformity). **(C)** Schematic summary of data presented in **(A,B)** showing significant bias in cilium emergence position (oriented pie slices color coded as in **A**) and axoneme orientation (arrows). Abbreviations as in Figure 2B ^∗^*p* < 0.05, ^∗∗^*p* < 0.01, ^∗∗∗^*p* < 0.005, ^****^p < 0.001. *N* = 4 independent specimens.

Preferential positioning of the cilium was assessed across zones of the developing humerus through measuring the distance of the cilium base from the center of the nucleus, scored for position within cell quadrants with respect to the major axes of the rudiment (Figures 1B, 3A). A wide range of distances from the center of the nucleus was observed across all zones (data not shown), however, there were significant regional differences in the preferred aspect of the cell from which the cilium emerged (Figure 3A). At both shoulder and elbow joints and the associated resting zones, the largest proportion of cilia were facing away from the joint line (distal and proximal quadrants for shoulder and elbow, respectively) (38–58%, *p* < 0.001). Interestingly, cilia in the proliferative and hypertrophic zones were also unevenly distributed: both with the highest proportion of cilia in the posterior quadrant (48%, *p* < 0.05; 37%, *p* < 0.05, respectively). In the proliferative zone a tiny proportion of cilia (4%) emerged from the distal aspect. These positional biases were also revealed through analysis of rose histograms which graphically represent degree of alignment (data not shown).

Next, bias in the orientation of the axoneme was assessed (Figure 3B). At the elbow, cilia tended to be oriented away from the joint (proximal). Interestingly, in the elbow resting zone, cilia were preferentially oriented both toward and away from the joint line (Kuiper’s test of uniformity; *p* < 0.01), but neither the shoulder resting zone nor joint had a significantly non-uniform distribution. Relatively few, very short cilia were visible in the hypertrophic zone, so were not included in analysis.

### Primary Cilia Are Shorter in Immobile, Muscleless Limbs of *Pax3*^*Spd/Spd*^ Embryos

**FIGURE 4:**
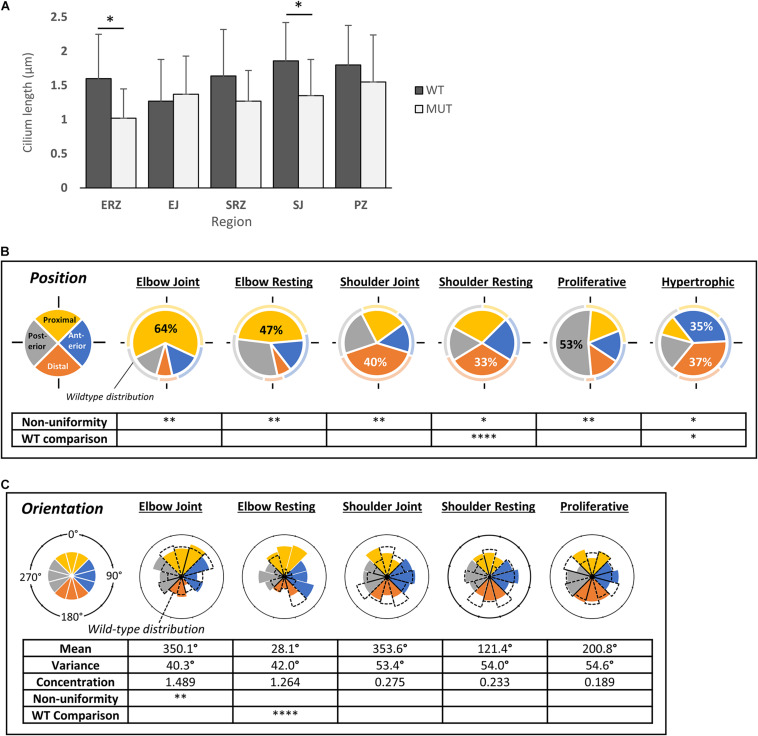
Cilia on immobile embryo (*Pax3*^*Spd/Spd*^) rudiment chondrocytes are shorter but have similar position of emergence and orientation compared to wildtype cilia. **(A)** Comparison of cilium length between wildtype (WT) and immobile mutant (*Pax3*^*Spd/Spd*^) (MUT) across different regions of the developing humerus at TS23, as indicated. Significance was determined by paired *t*-tests. **(B)** Pie charts of cilia emergence position on chondrocytes in different regions of the mutant humerus, where each slice represents the proportion of cilia located in the given quadrant (as illustrated in Figure 1B); color codes per named quadrant as shown on extreme left. The wildtype proportion in each quadrant for the same position is shown around each chart circumference for comparison. Significant differences from uniform distribution (non-uniformity) and from the wildtype distribution (WT comparison) are shown (chi-squared tests). **(C)** Rose histograms of cilium axoneme orientation on chondrocytes in different regions of the mutant humerus; for comparison the wildtype pattern is shown as dashed outlines. Angular mean and variance, concentration, and non-uniformity were calculated as for wildtype (Figure 3B). Values for WT (wildtype) comparison represent the results of Mardia-Watson-Wheeler/Uniform Scores tests, as calculated using the CircStats package in R. Significance values: ^∗^*p* < 0.05, ^∗∗^*p* < 0.01, ^****^p < 0.001. *N* = 4 independent wildtype specimens and *n* = 3 independent mutant specimens; except *n* = 3 and *n* = 2, respectively, for length measurements.

Several aspects of skeletal development in the humerus and associated joints of muscle-less embryos (e.g., *Pax3*^*Spd/Spd*^) are severely affected at TS23 (Kahn et al., 2009; Nowlan et al., 2010; Rolfe et al., 2014). Comparing cilia occurrence in the developing humerus in *Pax3*^*Spd/Spd*^ to wildtype littermates at this stage showed similar proportions of ciliated cells, with no significant differences between wildtype and mutant in any zones observed (Supplementary Table 1). Comparing cilium length across regions of the humerus, however, showed significant reduction in the mutant elbow resting zone (mean 1.02 ± SE 0.43 vs. 1.60 ± SE 0.65), and shoulder joint (mean 1.35 ± SE 0.53 vs. 1.86 ± SE 0.56) (Figure 4A). Although significance was not reached in other regions, there was an overall trend of a shorter mean cilium length. Cilia in the hypertrophic zone were excluded from statistical analyses because of their short length.

The biases seen in the positioning of the cilium on the side of the cell away from the shoulder and elbow joint lines in wildtype was replicated in the mutant (Figure 4B). At both the elbow joint and resting zone, the highest proportion of cilia was again in the proximal quadrant (47 and 64%, respectively, *p* < 0.001). Likewise, at the shoulder joint and resting zone, the highest proportion was in the distal quadrant (33%, *p* < 0.001 and 40%, *p* < 0.05, respectively), although there was a reduction in bias in the resting zone with a significant difference between mutant and wildtype in this respect. In the wildtype proliferative zone, the largest proportion of cilia was in the posterior quadrant; this was also observed in the mutant, with an even larger proportion of cilia in this quadrant (53% compared to 48% in the wildtype). However, in the mutant hypertrophic zone, the posterior bias seen in the wildtype is lost with predominant positioning on anterior and distal aspects.

Orientation of the axoneme was also assessed in mutant rudiments, showing that only the elbow resting zone was significantly different to wildtype (*p* < 0.0001), where the bi-modal distribution was lost in the mutant with instead a pattern not significantly different to a full range of orientations (Figure 4C).

## Discussion

Appropriate development of long bones and joints in the limb skeleton requires mechanical stimulation from movement, and while the molecular mechanisms involved are not known, the primary cilium represents a candidate mechanotransductor. To explore a link between mechanosensitivity of the developing skeleton and the primary cilium, we investigated the proportion of ciliated cells, and the length and position in 3D space of the cilium across tissue zones of the developing humerus and associated joints at E14.5 (TS 23) in the mouse embryo; these locations were previously identified as highly sensitive to mechanical input from movement at this developmental timepoint. Cilia were identified by double immunostaining using basal body and axoneme markers, followed by automated image processing for quantification of cilia properties, demonstrating a simple approach for integrated characterization of multiple aspects of cilia properties across tissues. We show that cilia were detected throughout the rudiment with distinctive characteristics within zones, including more cilia in the joint regions and resting zones compared to the proliferative and hypertrophic zones. Strikingly, at the shoulder and elbow joints, cilia emerged predominantly from the side of cells orientated away from the joint, while the proliferative and hypertrophic zones showed preferred positioning on the posterior aspect. To further investigate a link with mechanosensation we also compared patterns in rudiments of muscle-less limb mutants, to examine if cilia properties are altered when mechanical stimulation from movement is absent. Cilia at the mutant joints maintained their position, oriented away from the joint line, and while the proportion of ciliated cells was not significantly altered in the mutant, cilia were significantly shorter in joint zones with an overall trend of shorter cilia.

Primary cilia can act as both mechanical sensors and molecular signaling centers, integrating biophysical and biochemical pathways (Muhammad et al., 2012; Thompson et al., 2014; Elliott and Brugmann, 2019). Primary cilia were previously detected on mature articular cartilage (Farnum and Wilsman, 2011) and the growth plates of late developing and neonatal skeleton (McGlashan et al., 2007; Song et al., 2007; de Andrea et al., 2010; Ascenzi et al., 2011). Here, we show for the first time that primary cilia are present across zones of the early skeletal rudiment during the period of heightened sensitivity to embryo movement when immobile embryos show skeletal abnormalities (Kahn et al., 2009; Nowlan et al., 2010; Rolfe et al., 2018; Singh et al., 2018). Across zones, the highest mean proportion of cells with detectable cilia was in the shoulder resting zone (70%), but without significant differences among resting zones and joint zones; the joint regions are particularly sensitive to reduced movement. Similarly high occurrence rates of cilia were found within adjacent morphological processes (olecranon and coracoid) undergoing expansion at that stage; these regions were previously shown to be misshapen when movement is reduced or absent (Roddy et al., 2011; Sotiriou et al., 2019). Significantly lower proportions of ciliated cells were found in the hypertrophic zone and, not surprisingly, in the proliferative zone since chondrocyte expansion is associated with loss of cilia (Thompson et al., 2017). No differences were found in terms of occurrence between wildtype and mutant.

The length of the cilium is variable across different cell types and tissues (summarized in Dummer et al., 2016). Cilia measured here are relatively short compared to other cell types but consistent with chondrocyte studies (typically between 1–4 μm) (Poole et al., 2001; McGlashan et al., 2008; Ascenzi et al., 2011; Thompson et al., 2014; Martin et al., 2017). Cilia detected in the hypertrophic zone were extremely short (approx. 0.08–0.14 μm), to the extent that they could not be accurately analyzed using the current approach. It is possible that primary cilia are not functional on cells undergoing hypertrophy. Across other zones, elbow joint cilia were significantly shorter than proliferative zone and shoulder joint. A difference was seen in cilia length in the mutant, with reduced length in regions associated with both joints, and a trend toward reduced mean length across zones. Cilium length can be responsive to the mechanical and chemical signaling environment; for example, artificially elongated cilia on bone cells are more mechanosensitive (Ehnert et al., 2017; Spasic and Jacobs, 2017a). In response to compression or fluid flow, chondrocyte, tendon, and renal cilia shorten (Resnick and Hopfer, 2007; McGlashan et al., 2010; Gardner et al., 2011). Changes in cilia length have also been shown to alter Hh and Wnt cell signaling dynamics (Wann and Knight, 2012; McMurray et al., 2013; Thompson et al., 2014). The altered cilium length on chondrocytes of the developing humerus in the immobile embryo could contribute to the phenotype.

Defining spatial characteristics is important in addressing how the primary cilium might act as a sensory receptor. To achieve this, we examined the position of emergence of the ciliary axoneme on the cell and the orientation of projection relative to the long axis of the skeletal rudiment and show differential patterns between zones. There is striking polarization at the joint regions and their associated resting zones in terms of cilium emergence from the side of the cell facing away from the joint line (Figures 3A,C). This polarization pattern was generally maintained in the muscle-less mutant (Figure 4A). Contrastingly, we saw little polarization in terms of the direction in which the axoneme projects. The only significantly non-uniform pattern was seen in the elbow joint and its associated resting zone with an enrichment of direction away from the joint line (proximal) at the joint and a bidirectional preference (toward and away from the joint line) in the resting zone. Interestingly while the enrichment in orientation away from the elbow joint was maintained at the mutant elbow, the bidirectionality in the resting zone was lost (Figure 4B). Farnum and Wilsman (2011) also found similar polarization in the position of emergence but no polarization in directionality of the axoneme according to zone within mature equine articular cartilage. They found that the cilium emerged from the side of the cell away from the joint line in the superficial zone but tended to be bidirectional (oriented away from and toward the joint surface) in the deeper radiate zone.

Previous studies on chondrocytes within the later stage growth plate found that cells of the proliferative and prehypertrophic/hypertrophic zones tend to emerge either at the proximal or distal side of cells in a pattern postulated to reflect chondrocyte columnar organization (Haycraft and Serra, 2008; Farnum and Wilsman, 2011). In contrast, we found polarization in proliferative and hypertrophic zones toward the posterior of the humerus which rather recalls the orientation of cell division in the proliferative zone, prior to intercalation (Li and Dudley, 2009). Ascenzi et al. (2011) examined the disorganized growth plate in Hedgehog signaling-impaired mice, reporting corresponding changes in cilium orientation. It is also possible that polarization is related to the directionality of biophysical stimuli, as seen in the tendon where cilia are aligned to the primary loading direction (Donnelly et al., 2010) and in articular cartilage where polarization is more defined in load bearing regions (Farnum and Wilsman, 2011). In this context it is interesting to note that computational modeling predicts posterior and anterior peaks of dynamic biophysical stimuli in the diaphysis of the humerus at this stage of development (Nowlan et al., 2012) and that positional polarization is altered in the mutant hypertrophic zone (Figure 4B).

While the current study does not prove a role for primary cilia in sensation of movement-generated mechanical cues, the demonstration of cilia in region-specific patterns, during heightened developmental sensitivity to movement, highlights the capacity of cilia to contribute to skeletal tissue differentiation. The method used to assess cilia occurrence detects cilia by a double-positive immunolocalization signal and calculates this as a proportion of DAPI-positive nuclei within the ROI. It is possible that this is an underestimate if nuclei are captured and the cilium is outside the ROI, however, we believe this is minimized by using a threshold size for the detected DAPI signal and given that up to 80% of cells were found to be ciliated in some zones. Additionally, chondrocyte nuclei occupy a large proportion of the cell volume, minimizing the number of cilia which would be outside the ROI. Finally, this would not impact comparison across zones and between wildtype and mutant.

The analysis of more mutant specimens (*n* = 3) might have increased the significance levels in the differences observed, however, the current findings build a substantial profile of cilia characteristics across multiple sites in the developing rudiment, as well as an overall comparison with the muscle-less limb state. Higher resolution imaging, such as Electron Microscopy, could provide more detail on axoneme shape and orientation but the methodology developed here is valuable in offering a relatively simple approach for simultaneous assessment of multiple aspects of cilia properties across multiple territories.

Further exploration of the primary cilium in this context is required to understand its potential role in mechanoregulation of specific processes during skeletal development. We have previously characterized gene expression changes in the developing humerus under reduced mechanical stimulation (Rolfe et al., 2014) and additionally showed that territories of Wnt and BMP signaling at the developing joint are disturbed under immobilization (Rolfe et al., 2018; Singh et al., 2018). In this context it is intriguing that primary cilia potentially link mechanical and molecular signaling (Elliott and Brugmann, 2019; Bosakova et al., 2020). Primary cilia loss has been shown to upregulate canonical Wnt signaling, in some contexts (Corbit et al., 2008; Jiang et al., 2016; Patnaik et al., 2019). Ciliary activity might therefore restrain canonical Wnt activity (Lancaster et al., 2011) which is spatially restricted in a mechanosensitive manner in the developing rudiment (Singh et al., 2018). Cilia are also suggested to mediate switching between canonical and non-canonical Wnt signaling (Simons et al., 2005; Corbit et al., 2008); non-canonical Wnt/PCP signaling is required for convergent extension of proliferating chondrocytes in the growth plate, a process which is disorganized when primary cilia are disrupted (Koyama et al., 2007; Song et al., 2007) as well as under immobilization (Shwartz et al., 2012). Additionally, cilia may influence Hippo signaling (Saburi et al., 2008), linked through the actin cytoskeleton (Kim et al., 2015). We previously showed that the distribution of activated YAP is altered in immobile developing skeletal rudiments (Shea et al., 2020). Cilia could therefore link mechanical signals, intracellular signaling, and cytoskeletal architecture during skeletal development.

## Data Availability Statement

The raw data supporting the conclusions of this article will be made available by the authors, without undue reservation.

## Ethics Statement

The animal study was reviewed and approved by the Trinity College Dublin Bioresources Unit and Bioethics Committee, under license from the Irish Medicines Board/Health Products Regulatory Authority.

## Author Contributions

CS and PM contributed to conception and design of the study, and wrote, revised, and approved the manuscript. CS performed staining, image collection, and analysis. Both authors contributed to the article and approved the submitted version.

## Conflict of Interest

The authors declare that the research was conducted in the absence of any commercial or financial relationships that could be construed as a potential conflict of interest.

## Publisher’s Note

All claims expressed in this article are solely those of the authors and do not necessarily represent those of their affiliated organizations, or those of the publisher, the editors and the reviewers. Any product that may be evaluated in this article, or claim that may be made by its manufacturer, is not guaranteed or endorsed by the publisher.
