# Supplementary material for: The Primary Cilium on Cells of Developing Skeletal Rudiments; Distribution, Characteristics and Response to Mechanical Stimulation
Source: Front Cell Dev Biol. 2021 Aug 20;9:725018. doi: 10.3389/fcell.2021.725018 (PMC8418538; doi:10.3389/fcell.2021.725018)
Supplement: Supplementary file 1 [file Table_1.DOCX]

# SUPPLEMENTARY MATERIAL

**Supplementary Table 1.** Cilia occurrence (expressed as proportion of total cells) on chondrocytes in zones of the wildtype and mutant forelimb rudiments.

| **Occurrence** | | | | | | | | | |
| --- | --- | --- | --- | --- | --- | --- | --- | --- | --- |
|  |  | **Coracoid process** | **Olecranon process** | **Elbow  joint** | **Elbow  resting zone** | **Shoulder joint** | **Shoulder resting zone** | **Proliferative zone** | **Hypertrophic zone** |
| **Wildtype** | Mean ± SE | 0.68 ± 0.06 | 0.55 ± 0.08 | 0.54 ± 0.05 | 0.63 ± 0.04 | 0.54 ± 0.05 | 0.70 ± 0.06 | 0.42 ± 0.07 | 0.33 ± 0.08 |
|  | Range | 0.52 - 0.80 | 0.43 - 0.77 | 0.32 - 0.73 | 0.45 - 0.74 | 0.39 - 0.69 | 0.49 - 0.89 | 0.26 - 0.65 | 0.15 - 0.54 |
| **Mutant** | Mean ± SE | 0.73 ± 0.05 | 0.62 ± 0.14 | 0.6 ± 0.08 | 0.71 ± 0.04 | 0.55 ± 0 | 0.56 ± 0.05 | 0.37 ± 0.03 | 0.45 ± 0.05 |
|  | Range | 0.53 - 1.00 | 0.50 - 0.64 | 0.40 - 0.68 | 0.49 - 0.72 | 0.26 - 0.47 | 0.32 - 0.54 | 0.57 - 0.92 | 0.51 - 0.86 |
|  | p-value | 0.4078 | 0.1106 | 0.8877 | 0.5076 | 0.5311 | 0.2559 | 0.6634 | 0.5915 |
